# Rapid Measurement of Molecular Transport and Interaction inside Living Cells Using Single Plane Illumination

**DOI:** 10.1038/srep07048

**Published:** 2014-11-14

**Authors:** Per Niklas Hedde, Milka Stakic, Enrico Gratton

**Affiliations:** 1 Laboratory of Fluorescence Dynamics, Department of Biomedical Engineering, University of California, Irvine, CA, USA

## Abstract

The ability to measure biomolecular dynamics within cells and tissues is very important to understand fundamental physiological processes including cell adhesion, signalling, movement, division or metabolism. Usually, such information is obtained using particle tracking methods or single point fluctuation spectroscopy. We show that image mean square displacement analysis, applied to single plane illumination microscopy data, is a faster and more efficient way of unravelling rapid, three-dimensional molecular transport and interaction within living cells. From a stack of camera images recorded in seconds, the type of dynamics such as free diffusion, flow or binding can be identified and quantified without being limited by current camera frame rates. Also, light exposure levels are very low and the image mean square displacement method does not require calibration of the microscope point spread function. To demonstrate the advantages of our approach, we quantified the dynamics of several different proteins in the cyto- and nucleoplasm of living cells. For example, from a single measurement, we were able to determine the diffusion coefficient of free clathrin molecules as well as the transport velocity of clathrin-coated vesicles involved in endocytosis. Used in conjunction with dual view detection, we further show how protein-protein interactions can be quantified.

Single particle tracking (SPT) methods have provided spectacular insight into biomolecular movement within live cells^1^. Yet, highly detailed trajectories can only be obtained with spatially well separated, slowly moving molecules exhibiting a strong, stable signal. Fluorescence recovery after photobleaching (FRAP) is compatible with high particle concentrations and sufficiently fast if single point detection is applied^2^. Yet, the half-life time of recovery depends on the illumination geometry and the ratio of bleaching speed to recovery rate, which makes it difficult to obtain absolute diffusion coefficients. To unravel fast molecular motion, quantify particle concentrations and study blinking rates, fluorescence fluctuation spectroscopy (FFS) methods have become a popular choice^3^. The basis of FFS is a small observation volume that renders particle number fluctuations significant compared to noise-induced fluctuations in a fluorescence intensity time trace. In principle, if the sample is very thin (<1 μm) a simple epifluorescence configuration could be used. But for thick specimens due to high background from out-of-focus planes it is impossible to apply FFS methods to epifluorescence data except for samples containing very bright and sparse particles. This is not the case with a solution of, e.g., fluorescein or the cell interior in general. Therefore, most implementations of FFS use a single-point detection scheme that requires an expensive confocal or two-photon microscope with rather slow imaging speed (~1 frame/s). Also, interpretation of correlation curves can be tedious, especially in a noisy environment such as a living cell. Thus, a fast, camera-based and easy-to-interpret approach is highly desirable. To obtain a small observation volume, we used single plane illumination microscopy (SPIM)^4^,^5^. In SPIM, excitation and detection are decoupled by the use of two objective lenses arranged perpendicular to each other (Supplementary Fig. S1). A thin sheet of light emanating from the excitation objective limits fluorescence excitation to the focal plane of the detection objective anywhere within a three-dimensional sample. In order to facilitate sample handling, we implemented SPIM in an upright configuration (uSPIM) with both objectives dipping into the culture dish from the top at a 45° angle with respect to the sample plane^6^. Such a design is compatible with conventional, coverslip-based sample preparation (Supplementary Fig. S2,3). For FFS, the fluorescence signal needs to be captured over time. Correlation of the time trace at different lag times results in a decay curve that can be fitted with a model function to extract information about molecular dynamics. In camera-based microscopy, a fluorescence time trace is produced for every pixel which can be analyzed individually^7^,^8^,^9^. However, single pixel analysis faces a severe problem. With typical exposure times in the millisecond range, the temporal resolution is not sufficient to capture fast processes such as diffusion of small proteins or dye molecules (Supplementary Fig. S4). Instead, with camera data, it is much more effective to apply spatiotemporal image correlation spectroscopy (STICS)^10^.

## Results

### Measuring fast diffusion of small molecules in solution

**Figure 1:**
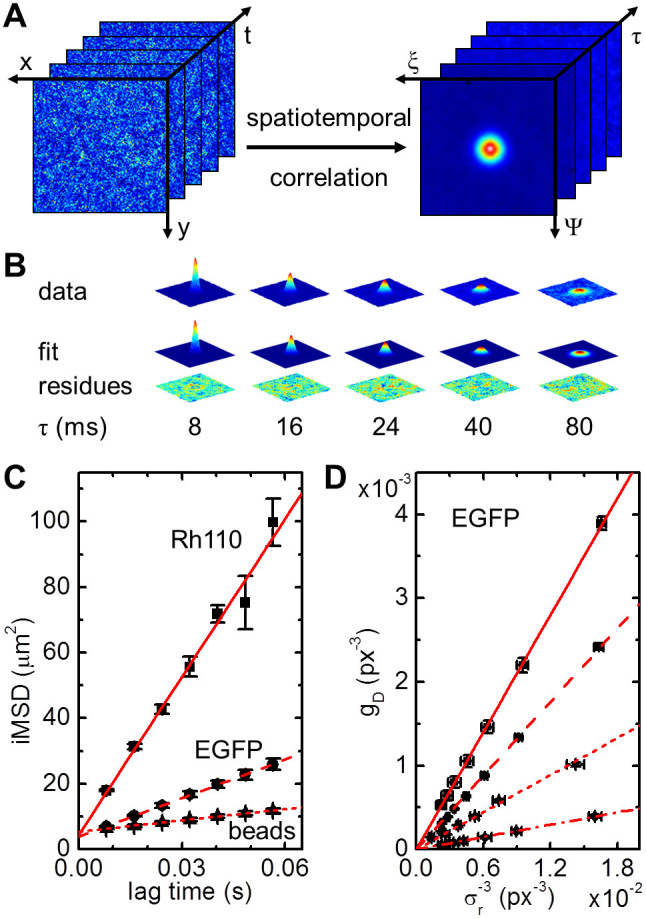
Solution measurements. (A) From a series of fluorescence images (left) the spatiotemporal correlation is calculated (right). (B) The data (here, EGFP in solution) can be approximated with a Gaussian function. The width of the peak corresponds to the particle *i*MSD. Free diffusion is indicated by a linear increase of the *i*MSD with the lag time, τ. (C) Average *i*MSD resulting from solution measurements of Rhodamine110 (squares, n = 6), EGFP (dots, n = 10) and 20 nm beads (triangles, n = 6). A linear fit of the data results in average diffusion coefficients of 400 ± 20 μm^2^s^−1^ for Rhodamine110 (solid line), 87 ± 7.5 μm^2^s^−1^ for EGFP (dashed line) and 29 ± 2.7 μm^2^s^−1^ for the beads (dotted line). (D) Average amplitude measured in EGFP solutions with different concentrations in the nM range (n = 5). Linear fits of the data results in average relative particle numbers inside the observation volume of 0.27 ± 0.01 (solid line), 0.43 ± 0.02 (dashed line), 0.85 ± 0.05 (dotted line) and 2.6 ± 0.1 (dash-dotted line). All errors stated are standard deviations.

The spatiotemporal correlations of an image series of immobile particles resembles the average shape of the particles convoluted with the microscope point spread function (PSF). For mobile molecules, the peak waist will broaden and the peak height will diminish with increasing lag time (Fig. 1A,B). In the case of free diffusion, the increase in peak width, i.e., the second order central moment, is proportional to the particle mean square displacement (MSD) (Eq. S2). By plotting the image MSD (*i*MSD) over the lag time, the average diffusion coefficient is determined by the slope of this linear relationship^11^. Notably, the slope is independent of the microscope PSF meaning that the calculation of the diffusion coefficient does not require any calibration of the instrument waist (Supplementary Fig. S5). Instead, the *i*MSD at zero lag time, i.e., the offset resulting from linear regression, points to the instrument PSF convoluted with the average particle size. To demonstrate that SPIM-*i*MSD is capable of precisely determining diffusion coefficients of fast moving molecules, we prepared nanomolar solutions of Rhodamine110, EGFP, and 20-nm red fluorescent beads. For each sample, SPIM image series were acquired and spatiotemporally correlated; the average *i*MSDs are plotted in Figure 1C. A comparison of the resulting diffusion coefficients with their literature values proves that SPIM-*i*MSD provides the correct numbers with high accuracy (Supplementary Table S1)^12^,^13^,^14^. In addition to dynamics, particle concentrations can be measured with FFS methods, too. The amplitude of the STICS function is inversely proportional to the average particle number inside the observation volume and, hence, the concentration. For free, three-dimensional diffusion, the amplitude depends linearly on the inverse of the third power of the peak waist with the slope being proportional to the inverse particle number (Eq. S3). This relation was verified with EGFP solutions of different concentrations (Fig. 1D). Note however, that these values are relative particle numbers. With an EMCCD camera as detector, the measurement of absolute particle numbers requires calibration^15^.

### 3D protein dynamics in living cells

**Figure 2:**
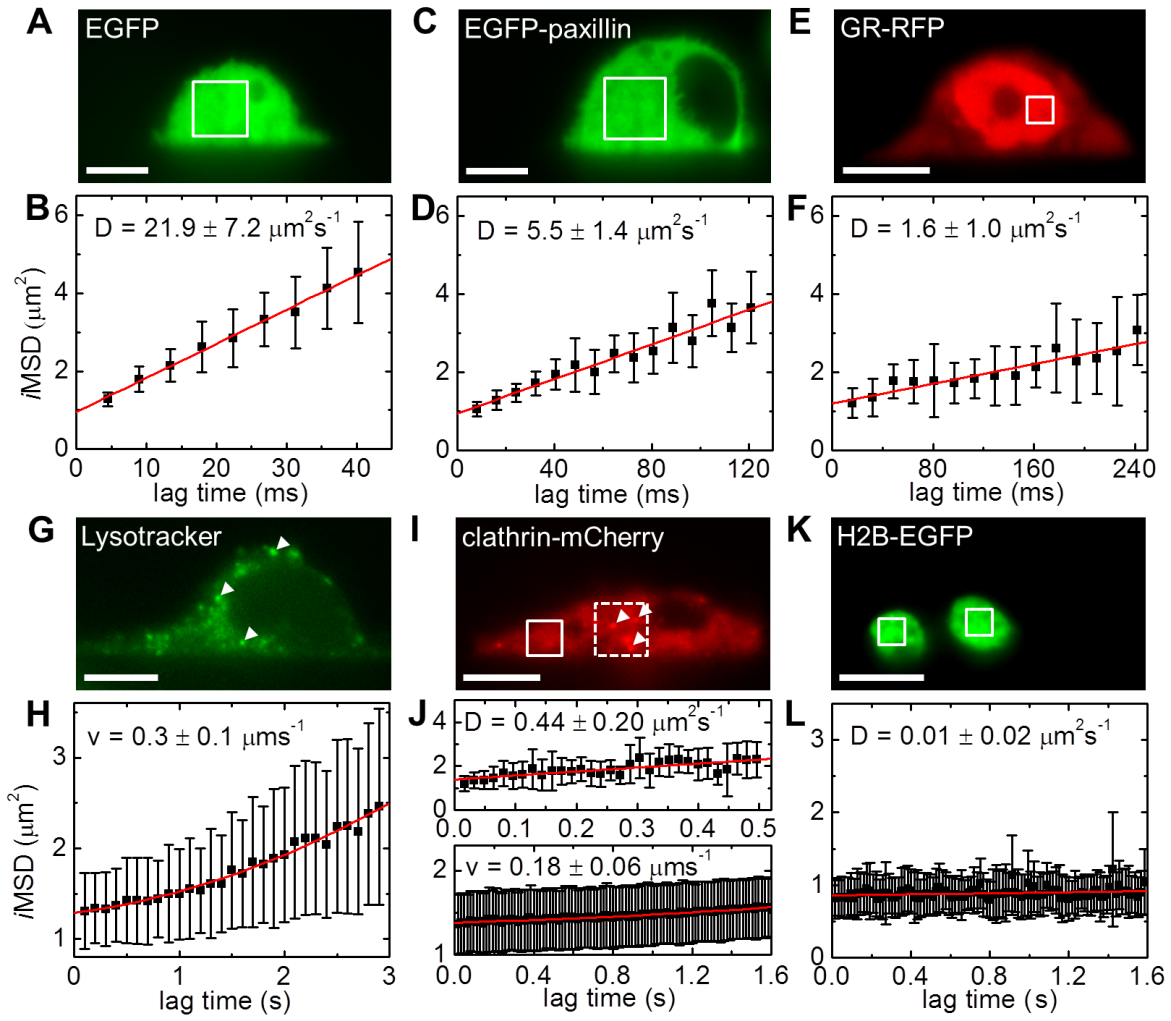
Molecular motion in live cells. (A) Fluorescence image of an EGFP expressing CHO-K1 cell. Image time series were recorded followed by STICS analysis in regions of 32 × 32 to 56 × 56 pixels (white box). (B) Average *i*MSD results in a diffusion coefficient of 21.9 ± 7.2 μm^2^s^−1^ (n = 13). (C) Fluorescence image of an EGFP-paxillin expressing CHO-K1 cell. STICS analysis in regions of 32 × 32 to 72 × 72 pixels (white box). (D) Average *i*MSD results in a diffusion coefficient of 5.5 ± 1.4 μm^2^s^−1^ (n = 11). (E) Fluorescence image of a GR-RFP expressing CHO-K1 cell. Image time series were subjected to STICS analysis in regions of 16 × 16 to 48 × 48 pixels (white boxes). (F) Average *i*MSD results in a diffusion coefficient of 1.6 ± 1.0 μm^2^s^−1^ (n = 6). (G) Fluorescence image of a CHO-K1 cell incubated with Lysotracker green. STICS data was analyzed in regions containing moving lysosomes (white arrows, 24 × 24 to 32 × 32 pixels). (H) A polynomial fit of the average *i*MSD returns an average lysosome velocity of 0.3 ± 0.1 μms^−1^ (n = 6). (I) Fluorescence image of a clathrin-mCherry expressing OK cell. STICS data was analyzed in two types of regions (24 × 24 to 64 × 64 pixels), one type exhibiting a homogeneous fluorescence signal (white box, solid line) and another type (white box, dashed line) containing slow moving vesicles (arrows). (J) *i*MSD of the two regions shown in panel (I). Linear fits of the data representing free clathrin (top) result in a diffusion coefficient of 0.44 ± 0.20 μm^2^s^−1^ (n = 7), whereas a polynomial fit to the data representing clathrin-coated vesicles (bottom) results in an average velocity of 0.18 ± 0.06 μms^−1^ (n = 6). (K) Fluorescence image of a H2B-EGFP expressing CHO-K1 cell. (L) A constant *i*MSD is obtained (D = 0.01 ± 0.02 μm^2^s^−1^, n = 8). Scale bars, 10 μm. All errors stated are standard deviations.

To evaluate the *in vivo* performance of SPIM-*i*MSD, we plated EGFP and EGFP-paxillin expressing Chinese hamster ovary (CHO) cells. Both proteins are known to diffuse freely in the cell cytoplasm, exemplary fluorescence images are shown in Figure 2A,C. For both samples, we recorded multiple SPIM image series and subjected them to *i*MSD analysis (Fig. 2B,D). Again, the resulting values agree very well with the diffusion coefficients found in literature (Supplementary Table S1). We also transfected CHO-K1 cells with plasmid encoding for the glucocorticoid receptor (GR) fused to RFP. Upon glucocorticoid (GC) treatment, GR molecules dimerize and translocate from the cell cytoplasm into the nucleus (Fig. 2E)^16^. Slow diffusion in the cell nucleoplasm was measured by means of SPIM-*i*MSD (Fig. 2F). Furthermore, as shown by Di Rienzo et al.^11^, the application of the *i*MSD method is not restricted to the case of free diffusion. For example, active transport can be easily identified on the basis of an increasing slope of the *i*MSD plot. By adding a component accounting for directed movement to the *i*MSD equation, the average particle velocity can be calculated (Eq. S4). To verify, we mimicked active transport by moving the sample stage with a known velocity during data acquisition within an EGFP solution (Supplementary Fig. S6). Within cells, lysosomes are known to be actively transported along the actin network. Figure 2G shows fluorescently labeled lysosomes inside live CHO-K1 cells (white arrows); a directed motion is immediately apparent from the curvature of the corresponding *i*MSD plot (Fig. 2H). Fitting a second order polynom to each dataset reveals an average lysosome velocity similar to results obtained with tracking experiments (Supplementary Table S1). Another mechanism involving active transport is endocytosis. Desirable molecules, such as nutrients, are recognized by antibodies decorating the plasma membrane. Clathrin triskelions floating in the cytoplasm bind via adaptor proteins, pull the membrane into a bud and form a vesicle that can be transported to various destinations including early endosomes. We imaged opossum kidney (OK) cells expressing a clathrin-mCherry fusion protein (Fig. 2I) and analyzed the data in two different types of regions. Unbound clathrin molecules exhibit a homogeneous fluorescence signal (white box) and, in such areas, free diffusion is indicated by a linear increase of the *i*MSD (Fig. 2J, top). Clathrin-coated vesicles, on the other hand, can be identified as bright, slowly moving dots (white arrows) and selective *i*MSD analysis indicates a directed motion (Fig. 2J, bottom; exemplary data of two measurements are shown in Supplementary Fig. S7). The resulting values are included in Supplementary Table S1. As a control, we transfected CHO-K1 cells with H2B-EGFP which is expected to be immobile (Fig. 2K,L). The acquisition parameters for all measurements are summarized in Supplementary Table S2.

### Studying protein interactions using cross-correlation analysis

**Figure 3:**
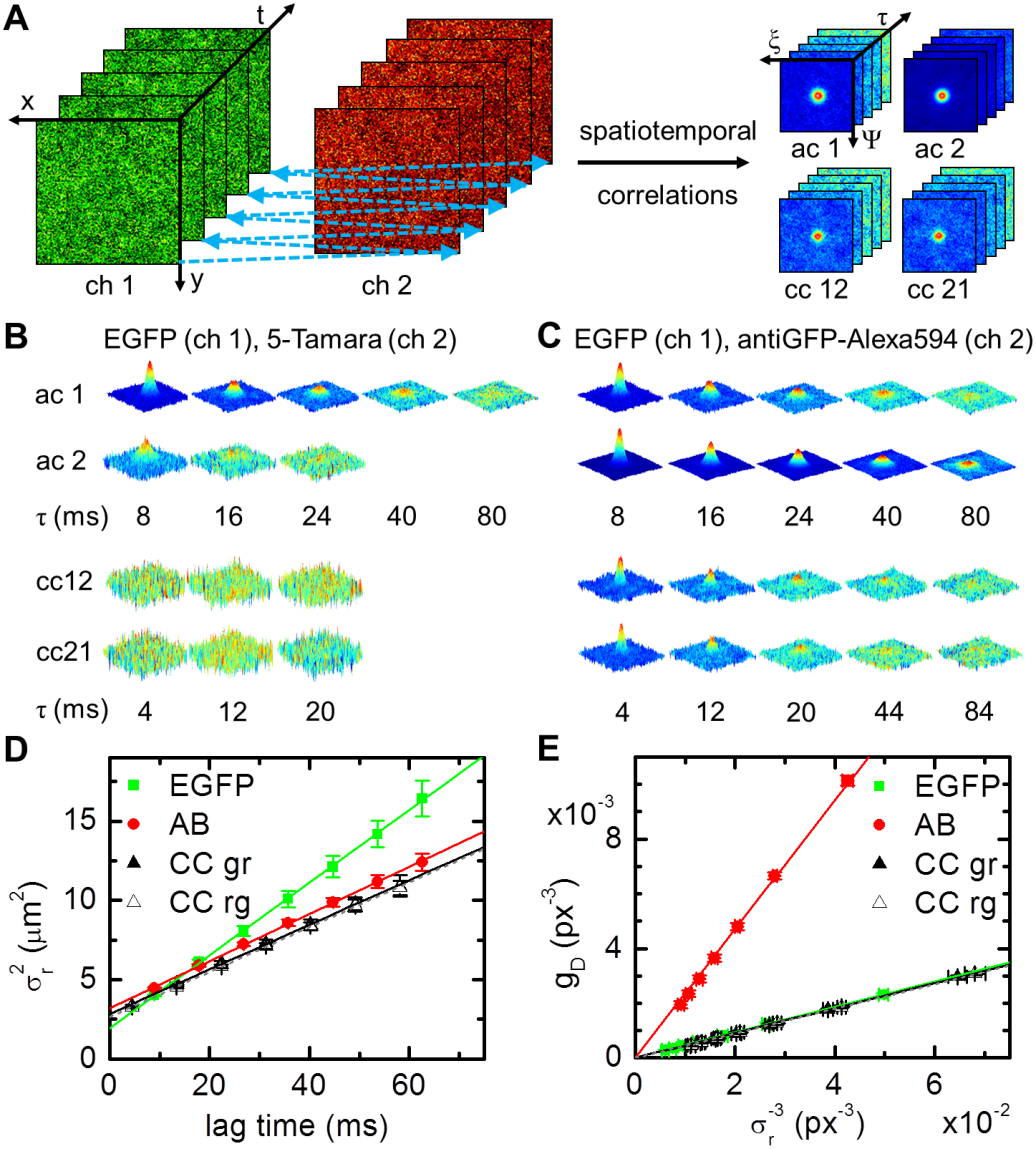
Principle of cc*i*MSD. (A) From a series of fluorescence images (left) acquired alternatingly in two channels, the autocorrelation in each channel as well as both cross-correlations are calculated (right). (B) STICS series obtained from a solution of EGFP (channel 1) and 5-Tamara (channel 2). The absence of any cross-correlation indicates no interaction between both molecules. (C) STICS series measured in a nanomolar solution of EGFP (channel 1) and antiGFP-Alexa594 (channel 2) mixed at a 4:1 ratio. Due to binding of the antibody to EGFP there is cross-correlation. (D) *i*MSD plots of channel 1, channel 2, and both cross-correlations (n = 7). Linear fitting of the data results in average diffusion coefficients of 57 ± 5 μm^2^s^−1^ (channel 1), 37 ± 3 μm^2^s^−1^ (channel 2), and 35 ± 3 μm^2^s^−1^ (cross-correlation). (E) Respective correlation amplitude plots and linear fits resulting in average particle numbers within the observation volume of 1.09 ± 0.03 for free EGFP, 0.007 ± 0.005 for free antiGFP-Alexa594, and 0.260 ± 0.003 for the bound complex, indicating that all antibodies are bound to EGFP molecules. All errors stated are standard deviations.

Two-color cross-correlation FFS (ccFFS) experiments have revolutionized studies of molecular interactions^17^. Due to the limited spatial resolution of the instrument, colocalization studies are prone to misconceive close proximity as interaction, while only binding can give rise to non-zero cross-correlation amplitudes. We fitted our SPIM setup with a dual view, splitting the fluorescence into a green and a red channel imaged side-by-side onto the same camera chip (Supplementary Fig. S1,8). An alternating excitation (ALEX) scheme was used to exclude any crosstalk between both channels (Fig. 3A). To verify this approach, we imaged a solution containing non-interacting EGFP and 5-tamara fluorophores; no cross-correlation was detected (Fig. 3B). As an example of protein binding, we imaged EGFP molecules mixed with antiGFP-Alexa594 antibodies (ABs) at a 4:1 ratio; the presence of a cross-correlation amplitude confirms molecular interaction (Fig. 3C). The corresponding *i*MSDs and amplitudes are plotted in Figure 3D,E, quantification shows that all ABs are bound to EGFP molecules. Interestingly, since all camera pixels are captured simultaneously, there is no need for precise lateral alignment of both detection channels (Supplementary Fig. S9).

### Protein interactions in living cells

**Figure 4:**
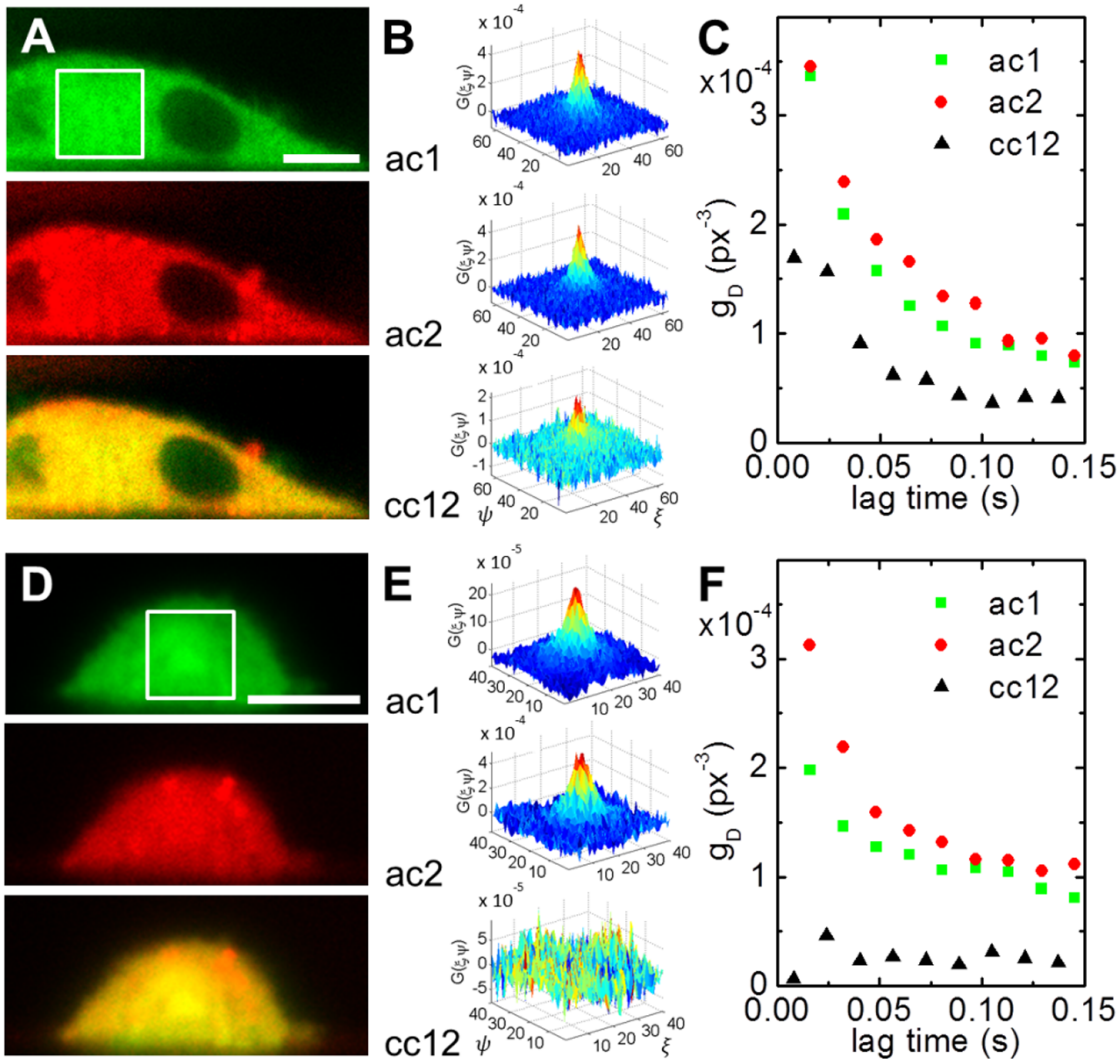
cc*i*MSD in live cells. (A) Fluorescence images of an EGFP-paxillin-mCherry expressing CHO-K1 cell (top, green channel; middle, red channel; bottom, overlay). A series of SPIM images was acquired alternatingly in both channels. (B) The resulting spatiotemporal autocorrelations in each channel (at 16 ms lag time) as well as the cross-correlation (at 8 ms lag time) are shown for the region marked by the box in panel (A). (C) Amplitudes returned from Gaussian fitting of each STICS series plotted over the lag time (squares, green channel; dots, red channel; triangles, cross-correlation). (D) Fluorescence images of an EGFP-paxillin and mCherry-paxillin expressing CHO-K1 cell. (E,F) The absence of any cross-correlation indicates no binding between both fusion proteins. Scale bars, 10 μm.

To demonstrate the capability of SPIM-cc*i*MSD to measure molecular interactions in living cells, we transfected CHO-K1 cells with a plasmid encoding for an EGFP-paxillin-mCherry fusion protein (Fig. 4A). As expected, the cross-correlation shows concurrent movement of green and red marker proteins (Fig. 4B,C). As a control, CHO-K1 cells expressing both EGFP and mCherry were subjected to SPIM imaging. While the fluorescence images show perfect colocalization (Fig. 4D), the absence of any cross-correlation proves that there is no interaction between both proteins (Fig. 4E,F).

## Discussion

Due to its high speed, low phototoxicity and cost-effectiveness, camera-based SPIM has revolutionized 3D imaging and started to replace single point scanning microscopy. However, in a single pixel, detection with a camera can never be as fast as with a single point detector, preventing studies of rapid, single molecule dynamics. In this work, we have demonstrated how the abundance of spatial information obtained with SPIM can be used to recover molecular movement in 3D; we were able to study processes ranging from slowly moving vesicles in cells (~0.2 μm^2^s^−1^) to fast diffusing dye molecules in solution (~400 μm^2^s^−1^) spanning a timescale of almost five orders of magnitude. In principle, raster image correlation spectroscopy (RICS)^18^,^19^ based on single-point scanning, can provide the same information, but data acquisition takes longer (>1 min) and requires oversampling of the PSF resulting in a limited field of view (~10 × 10 μm^2^). On the other hand, by application of stimulated emission depletion, RICS can provide insight into submicron sized regions of interest^20^. However, the field of view with STED-RICS is even more restricted and STED compatible dyes are demanded. Also, depending on the type of particle motion, fitting of RICS data with the correct model function can be difficult. On the contrary, different types of motions such as free diffusion, transient trapping and/or active transport are easily distinguishable by means of the *i*MSD plot and their quantification does not require any reference measurements. Likewise, molecular interactions, which are hard to tackle by means of dual color SPT or FRAP, can be studied with cc*i*MSD facilitated by not demanding perfect lateral alignment of both detection channels. Also, number and brightness (N&B) analysis can be used as a complimentary technique since it can be applied to the same data acquired for *i*MSD analysis. Since *i*MSD analysis has been included in commercial software and does not require any special modifications of the setup or sample, the SPIM-(cc)*i*MSD method is immediately applicable to any existing SPIM system equipped with a high numerical aperture detection lens.

## Methods

### Solution measurements

For the single channel experiments, nanomolar solutions of Rhodamine110, EGFP and 20-nm red fluorescent beads in water were prepared. Green fluorescence was excited at 488 nm at 700 μW while red fluorescence was excited with 561 nm at 150 μW, both measured before entering the objective lens.

For the dual channel cross-correlation experiments, a mixture of EGFP and antiGFP-Alexa594 (Sigma-Aldrich, St. Louis, MO, USA) was prepared at a ratio of 4:1 and diluted in water to a nanomolar concentration. Green and red fluorescence were excited with 800 μW of 488-nm light and 900 μW of 561-nm light, respectively. An alternating excitation scheme was used to avoid crosstalk between the green and red detection channels. For each measurement, we acquired 8,192 images of 64 × 64 pixels at 4.5 ms exposure time with a pixel size of 736 nm.

### Cell sample preparation

Strips of 2 mm × 17 mm were cut from 30 × 24 mm No 1.5 coverslips (Fisher Scientific, Waltham, MA, USA) with a diamond tipped pen. Several of these strips were placed in a 35 mm cell culture dish and coated with fibronectin prior to plating and transfecting the cells. For fluorescence imaging, single strips were transferred to the imaging dish (Supplementary Fig. S2) filled with phosphate-buffered saline (PBS).

Chinese hamster ovary (CHO-K1) cells stably expressing either enhanced green fluorescent protein (EGFP) or paxillin-EGFP were cultured in a humidified, 5% CO_2_ atmosphere at 37°C in Dulbecco's Modified Eagle Medium (DMEM)/Nutrient Mixture F-12 (Life Technologies, Rockville, MD) supplemented with 10% fetal bovine serum (FBS), and 0.5 mg/mL geneticin (G418) to maintain selection of transfected cells. Nontransfected CHO-K1 cells were cultured in DMEM/Nutrient Mixture F-12 supplemented with 10% FBS, 1% (v/v) and penicillin/streptomycin.

For fluorescence imaging of lysosomes, these cells were incubated for 1 h with 50 nM LysoTracker Green DND-26 (Life Technologies). For imaging of clathrin, GR, H2B and EGFP-paxillin-mCherry, the cells were transfected using Lipofectamine 2000 according to the manufacturer's instructions (Life Technologies). Generally, 1 μg of plasmid (diluted with PBS) was incubated with 5 μl of Lipofectamine for 30 min and added to the cell dish containing fully supplemented media. The cells were maintained in a humidified, 5% CO_2_ atmosphere at 37°C and used within 48 h. For the co-transfection experiments with EGFP and mCherry, the CHO-K1 cells stably expressing EGFP-paxillin were transiently transfected with plasmid encoding for mCherry. Translocation of GR-RFP to the nucleus was induced by incubating the cells with medium containing 1 μM of dexamethasone for 20 min.

### SPIM imaging and data processing

Fluorescence images were acquired with a home-built SPIM setup (Supplementary Fig. S1–3) running Micro-Manager (available at www.micro-manager.org). STICS correlation and *i*MSD analysis were performed with SimFCS (available at www.lfd.uci.edu) as well as custom Matlab (MathWorks, Natick, Massachusetts, USA) scripts. Data was visualized with SimFCS, Matlab and Origin (OriginLab, Northampton, USA).

### Data analysis

Before spatiotemporal correlation of the data, a potential immobile fraction was substracted. For free diffusion, as described in Di Rienzo et al., the spatiotemporal correlation *G_D_*(*ξ*,*ψ*,*τ*) at lag time τ, can be modeled with a Gaussian, 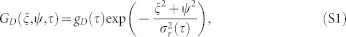
with pixel lags, *ξ* and *ψ*, along the *x* and *y* axes. The width, *σ_r_*(*τ*), represents the mean-square displacement of the particles within the image (*i*MSD), 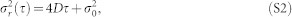
with the diffusion coefficient, *D*, which can be recovered from the slope. The *i*MSD at time zero, 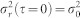, represents the convolution of the average particle size with the instrumental waist. For 3D diffusion, the amplitude, *g_D_*(*τ*), is given by 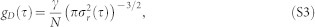
where *N* is the average number of particles inside the observation volume and *γ* = 0.35 a correction factor accounting for the contrast of the volume. Plotting the amplitude, *g_D_*(*τ*), against the *i*MSD, 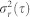, the average particle number N can be obtained.

In the case of active transport, there is an additional broadening of the correlation peak. This can be accounted for by adding a velocity term, *v*^2^*τ*^2^, to equation (2), 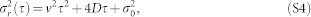
the average speed of the particles can be recovered.

For the cross-correlation experiments, there is a mixture of free green fluorescent particles *N_g_*, free red fluorescent particles *N_r_* and the bound complex *N_gr_*, which emits both green and red fluorescence assuming negligible photobleaching, no reaction-induced quenching or fluorescence enhancement, and the absence of particle exchange. Consequently, the average number of particles measured in the green channel, *N*_1_, is the sum of both free and bound green particles resulting in 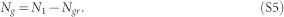
The same superposition applies to the average number of red particles, *N*_2_, yielding 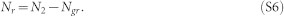
The particle number resulting from the cross-correlation function, *N*_12_, is directly proportional to the average number of dual labeled complexes, *N_gr_*. Assuming similar observation volumes for the green and the red channel we get 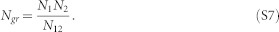


## Author Contributions

E.G. and P.N.H. designed the experiments. P.N.H. built the instrument, collected and analyzed data. M.S. cultured cells and prepared samples. E.G. developed software for data analysis. P.N.H. and E.G. wrote the manuscript.

## Supplementary Material

Supplementary Information Supplementary Information
