# Supplementary material for: Rapid Measurement of Molecular Transport and Interaction inside Living Cells Using Single Plane Illumination
Source: Sci Rep. 2014 Nov 14;4:7048. doi: 10.1038/srep07048 (PMC4231332; doi:10.1038/srep07048)
Supplement: Supplementary Information [file srep07048-s1.doc]

**Supporting Information**

**Rapid Measurement of Molecular Transport and Interaction inside Living Cells Using Single Plane Illumination**

Per Niklas Hedde^#^, Milka Stakic^#^ & Enrico Gratton^#^

^#^Laboratory of Fluorescence Dynamics, Department of Biomedical Engineering, University of California, Irvine, CA, USA.

**Supporting Figures and Tables.**

| **Figure S1** | Schematic of the uSPIM setup. |
| --- | --- |
| **Figure S2** | Sample mounting. |
| **Figure S3** | Picture of uSPIM setup. |
| **Figure S4** | Single-point FCS analysis of camera image data. |
| **Figure S5** | *i*MSD Analysis with different pixel sizes. |
| **Figure S6** | *i*MSD of an EGFP solution moved at different velocities. |
| **Figure S7** | *i*MSD analysis of Clathrin-coated vesicles. |
| **Figure S8** | Lightsheet thickness. |
| **Figure S9** | Cross-correlation with offset images. |
| **Table S1** | Overview of diffusion coefficients and velocities determined by SPIM-*i*MSD and comparison with literature values. |
| **Table S2** | Summary of the SPIM data acquisition parameters. |

**Supporting Figures**

**
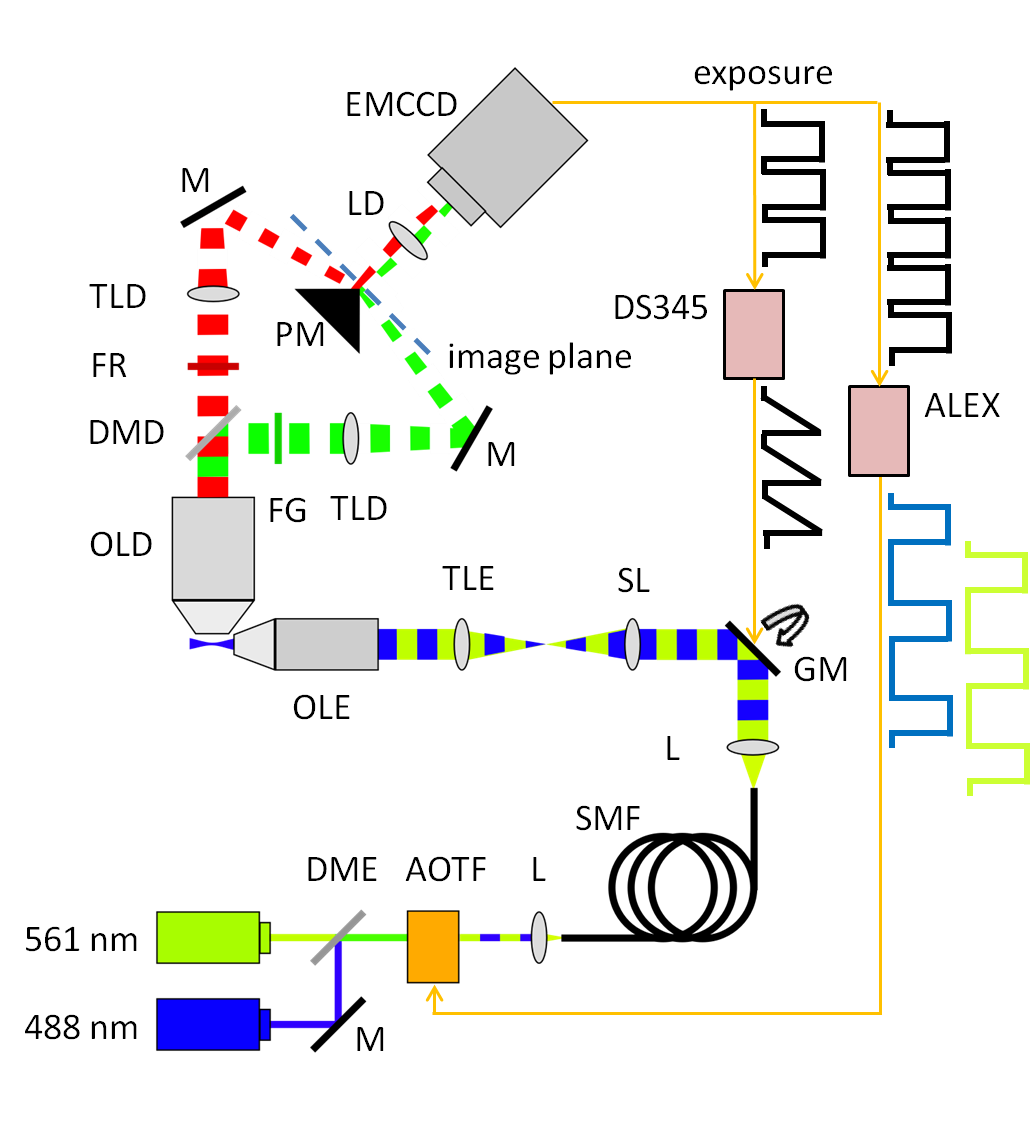
**

**Figure S1.** Schematic of the uSPIM setup. For exciting green and red fluorescence, a 488-nm laser diode (488nm, ISS, Champaign, IL, USA) and a 561-nm solid state laser (CL561-150, CrystaLaser, Reno, NV, USA) are combined via a long pass dichroic mirror, DME (515 LP, Chroma, Bellows Falls, VT, USA). Passing through an acousto optical tunable filter, AOTF, (AOTFnC-400.650, AA Opto-Electronic, Orsay, France), the laser profiles are spatially cleaned by focusing the light into a single mode fiber, SMF. After exiting the fiber and subsequent collimation, the excitation beam passes a scanning system consisting of a galvo mirror, GM (GVSM002, Thorlabs, Newton, NJ, USA), a scan lens, SL (#49-356, Edmund Optics, Barrington, NJ, USA), and a tube lens, TLE (#49-362, Edmund Optics, Barrington, NJ, USA), before the light is focused by an objective lens, OLE (CFI Plan Fluor 10XW, Nikon, Melville, NY, USA). The focal plane of the detection objective, OLD (Scaleview 25x, Olympus, Center Valley, PA, USA), is illuminated by scanning the excitation lobe across the entire field of view once per exposure cycle using a voltage ramp created by a function generator (DS345, SRS, Sunnyvale, CA, USA) synchronized to the camera. Fluorescence light is split by a long pass dichroic mirror (FF560, Semrock, Rochester, NY, USA). The transmitted portion of the beam is filtered by a band pass, FR (605/70 ET, Chroma, Bellows Falls, VT, USA), and imaged onto a knife-edge prism mirror, PM (MRAK25-P01, Thorlabs, Newton, NJ, USA), via a tube lens, TLD (#47-737, Edmund Optics, Barrington, NJ, USA). Spectrally cleaned by a different band pass filter, FG (FF03-525/50, Semrock, Rochester, NY, USA), the reflected portion is imaged onto the other face of the prism mirror by a symmetric arrangement. Both channels are imaged onto the chip of an EMCCD camera (Evolve 512, Photometrics, Tucson, AZ, USA). For cross-correlation measurements, fluorescence is alternately excited (ALEX) to avoid crosstalk between both detection channels.


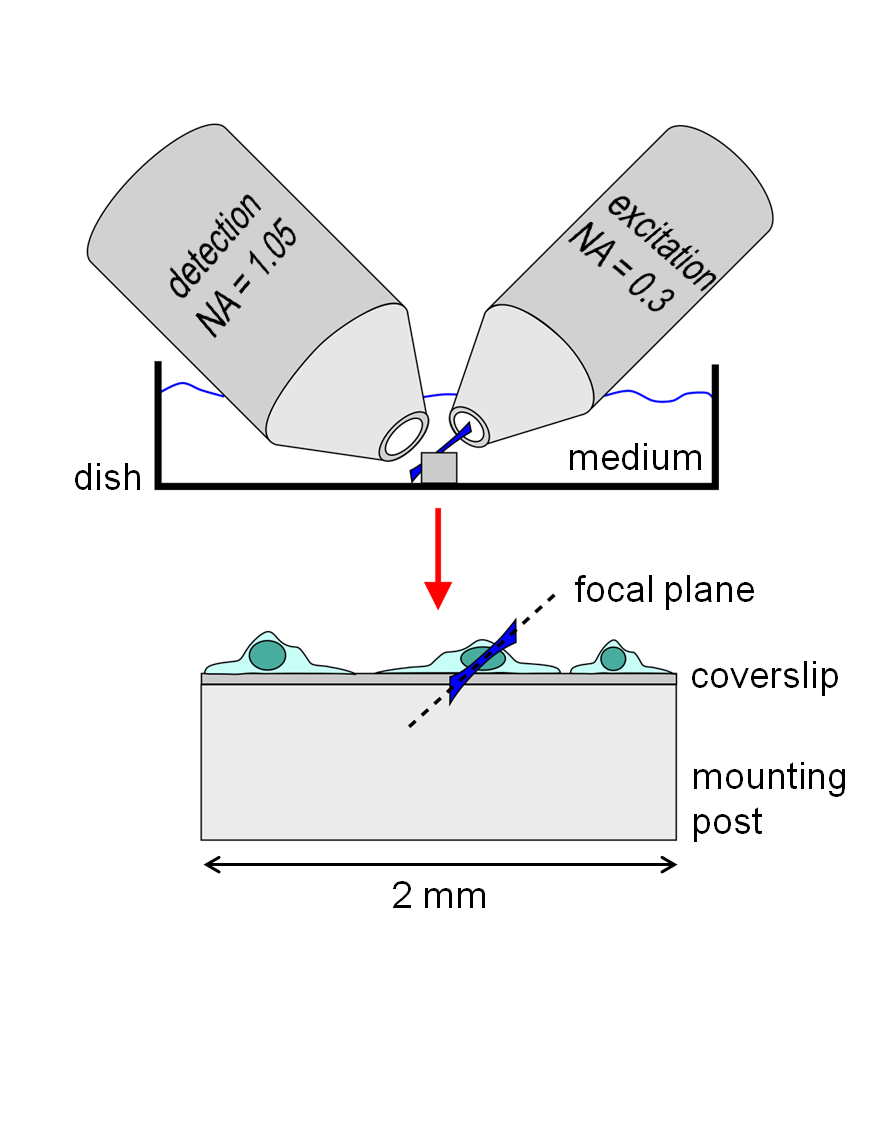


**Figure S2.** Sample mounting. Since the acceptance angle of the high-NA detection lens is less than 45°, the sample needs to be slightly elevated into the space between both objective lenses. Thus, for cell imaging, a two millimeter wide mounting post is glued to the bottom of a 60 mm tissue culture dish and holds the cells grown on 2 mm × 17 mm coverslips.

**
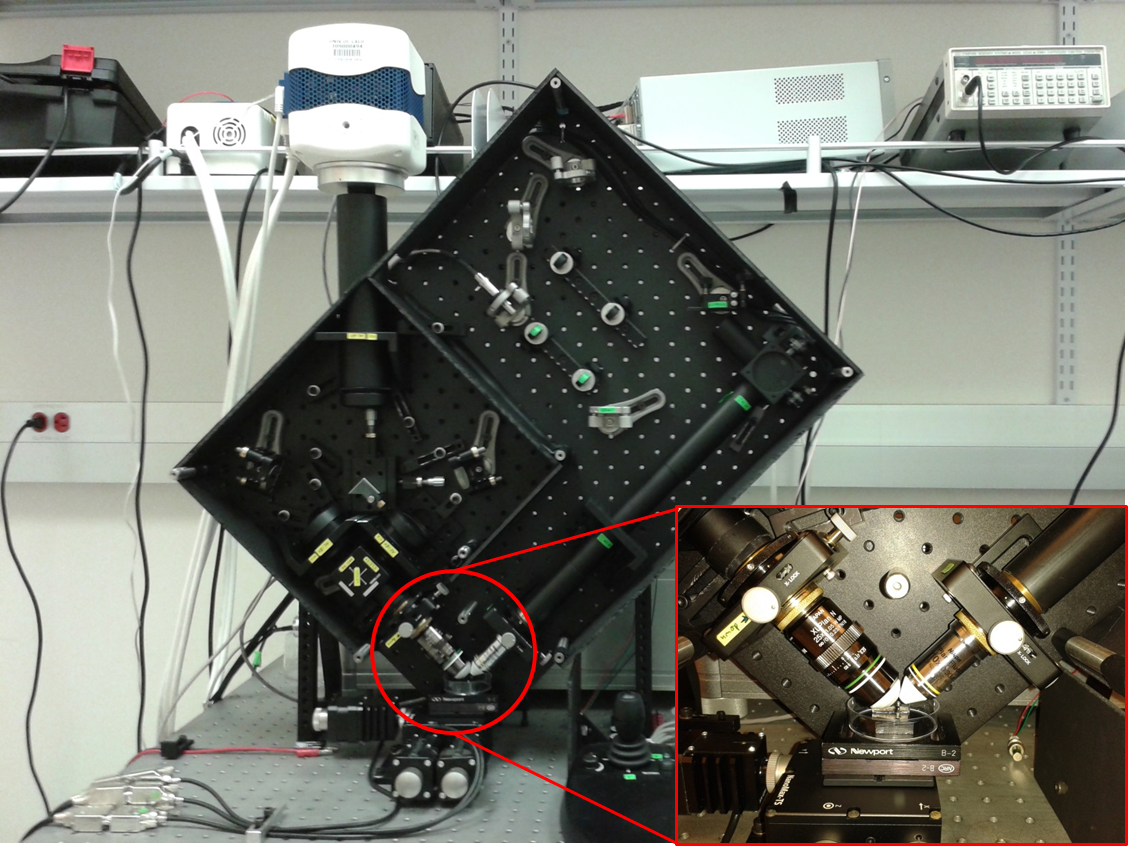
**

**Figure S3.** Picture of uSPIM setup. Picture of the uSPIM setup as described in Supplementary Fig. S1. The inset shows the two objective lenses dipping into the sample dish as described in Supplementary Fig. S2.


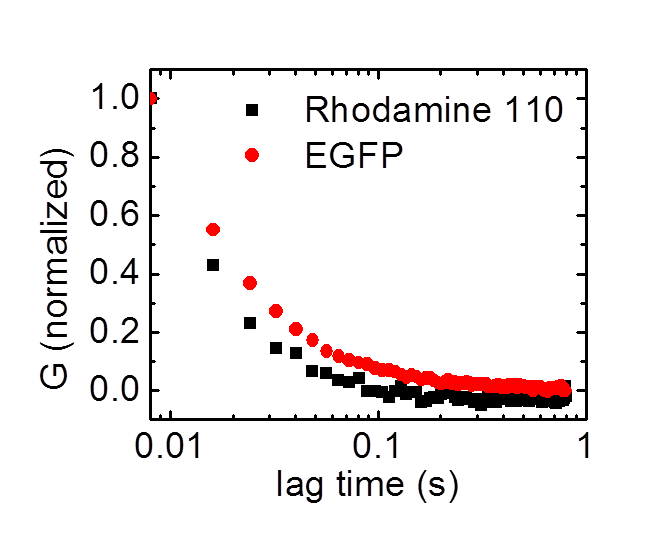


**Figure S4.** Single-point FCS analysis of camera image data. Nanomolar solutions of Rhodamine110 and EGFP were prepared and subjected to SPIM imaging (128 × 128 pixels) with a camera exposure time of 8 ms. The temporal autocorrelation was calculated for each pixel and averaged. The normalized amplitude is plotted over the lag time in the graph above. It can be seen that only the last portion of the correlation decay is captured since the diffusion time of both molecules exceeds the temporal resolution of the camera. Hence, a reliable fit of the data is not possible.

**
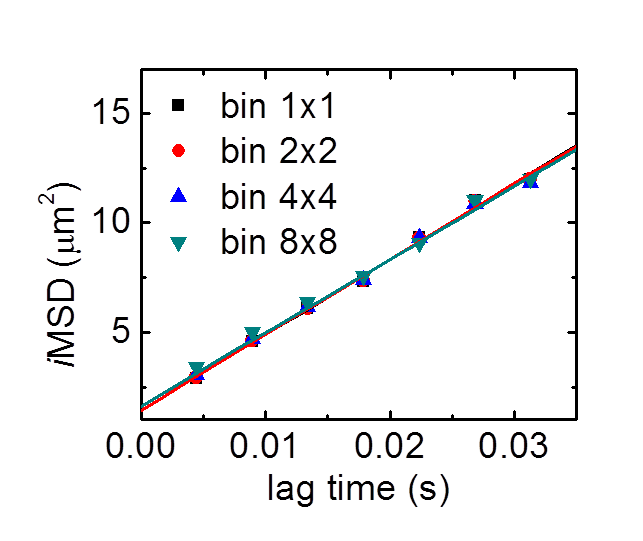
**

**Figure S5.** *i*MSD analysis with different pixel sizes. A 5-nM EGFP solution was prepared and a time series of 8,192 SPIM images was acquired with 4.5 ms exposure time. The images were differently binned yielding pixel sizes of 0.184 µm (1×1), 0.368 µm (2×2), 0.736 µm (4×4) and 1.472 µm (8×8) followed by *i*MSD analysis. There is only minimal influence on the resulting diffusion coefficients (86.6 µm^2^s^-1^, 86.1 µm^2^s^-1^, 83.6 µm^2^s^-1^ and 81.0 µm^2^s^-1^). Note a slight increase of the *i*MSD offset with increasing pixel size due to the loss in spatial resolution.

**
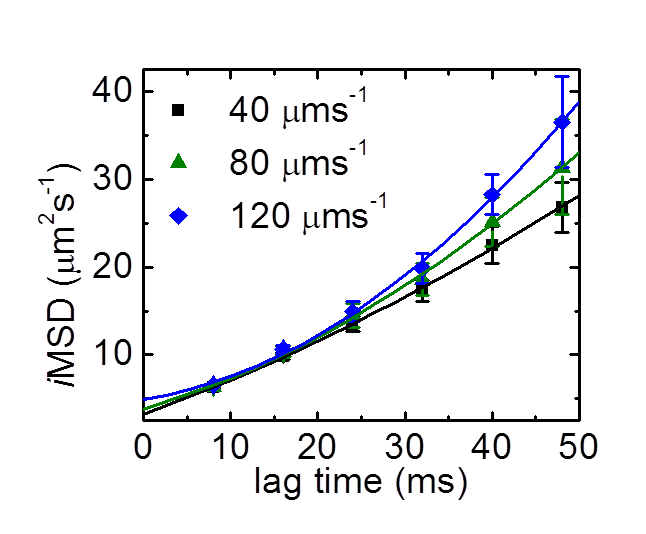
**

**Figure S6.** *i*MSD of an EGFP solution moved at different velocities. An EGFP solution (5 nM) was prepared and subjected to SPIM imaging with an exposure time of 8 ms. During the acquisition of each image time series, the stage was moved at velocities of 40 µms^-1^, 80 µms^-1^ and 120 µms^-1^ to mimic active transport. The velocities returned by *i*MSD analysis are 51 ± 13 µms^-1^, 71 ± 32 µms^-1^ and 100 ± 20 µms^-1^ (n = 7).

**Figure S7.** *i*MSD analysis of Clathrin-coated vesicles. Data from two, representative measurements is shown. Linear (blue) and polynomial fitting (red) was performed. As expected, the equation for active transport provides a better fit.


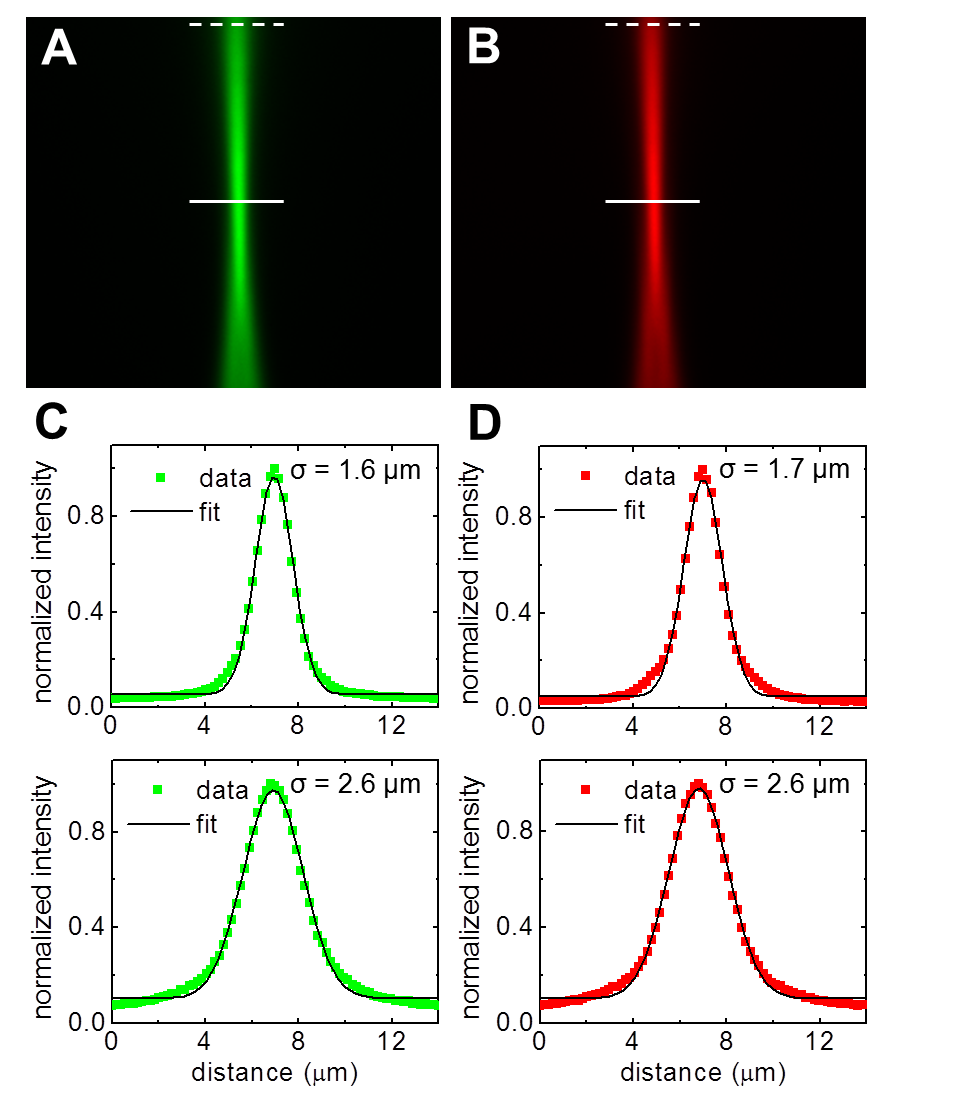


**Figure S8.** Lightsheet thickness. The light sheet is created by scanning the excitation lobe across the field of view. The thickness of the light sheet can be obtained by parking the beam and measuring the profile of the lobe. (A,B) Fluorescence images of the light sheet measured in an EGFP/5-Tamara solution using 488-nm/561-nm excitation (a). (C,D) Approximating the intensity profile with a Gaussian, the thickness of the light sheet (e^-2^) ranges from 1.6 µm (green channel) and 1.7 µm (red channel) in the center (solid line in panel A,B) to 2.6 µm towards the edges (dashed line in panels A,B).


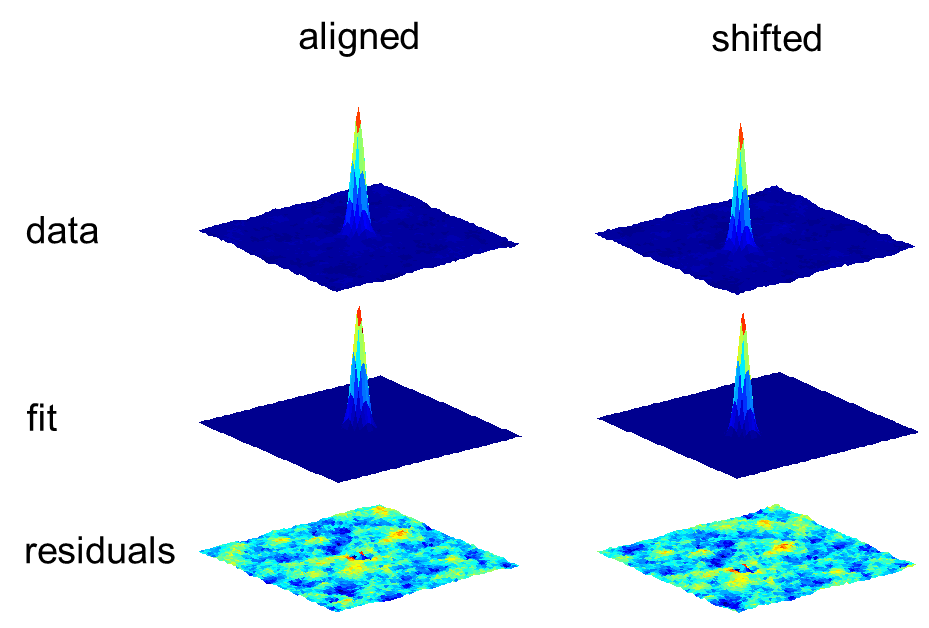


**Figure S9.** Cross-correlation with offset images. In cross-correlation fluctuation spectroscopy based on sequential single-point detection it is essential that both foci are perfectly overlapping. Any misalignment will significantly affect the correlation amplitude. On the contrary, with camera-based detection, the entire field of view is captured at the same time. Thus, any misalignment within the observation plane will only affect the position of the cross-correlation peak but not its amplitude or width. The left column shows the cross-correlation obtained with aligned detection channels from a solution of EGFP and antiGFP-Alexa594. A Gaussian fit to the data results in a width of 3.29 µm^2^ and an amplitude of 7.61 × 10^-3^ µm^-3^. The right column shows the same data but with the red image shifted by 5 and 9 pixels in *x* and *y* direction with respect to the green channel. Gaussian fitting gives almost the same values, that is 3.24 µm^2^ for the width and 7.57 × 10^-3^ µm^-3^ for the amplitude, respectively.

**Supporting Tables**

**Table S1.** Overview of diffusion coefficients and velocities determined by SPIM-*i*MSD and comparison with literature values.

| Molecule | Sample | Method | *D* (μm^2^s^-1^) | *v* (μms^-1^) | *T* (°C) | Reference |
| --- | --- | --- | --- | --- | --- | --- |
| *In vitro* | | | | | | |
| Rhodamine110 | Water | *i*MSD | 400 ± 20 | - | 25 | This work |
|  |  | PFG-NMR | 430 ± 30 |  | 22.5 | [^1^](#_ENREF_1) |
| EGFP | Water | *i*MSD | 87 ± 7.5 | - | 25 | This work |
|  |  | sFCS | 94 |  |  | [^2^](#_ENREF_2) |
|  |  | FCS | 93 ± 4* |  | 22 | [^3^](#_ENREF_3) |
| 20-nm beads | Water | *i*MSD | 29 ± 2.7 | - | 25 | This work |
| *In vivo* | | | | | | |
| EGFP | CHO-K1 | *i*MSD | 22 ± 7.2 | - | 25 | This work |
|  |  | RICS | 21 ± 5 |  |  | [^4^](#_ENREF_4) |
| EGFP-paxillin | CHO-K1 | *i*MSD | 5.5 ± 1.4 | - | 25 | This work |
|  |  | RICS | 3.1­­‒8.3 |  |  | [^5^](#_ENREF_5) |
| Lysosomes | CHO-K1 | *i*MSD | - | 0.3 ± 0.1 | 25 | This work |
|  | BS-C-1 | SPT |  | 0.4 ± 0.2 | 24, 37 | [^6^](#_ENREF_6) |
| GR-RFP | CHO-K1 | *i*MSD | 1.6 ± 1.0 | - | 25 | This work |
| GFP-GR | MA3617 | FRAP | 1.2 | - | NA | 7 |
| Clathrin-mCherry (free) | OK | *i*MSD | 0.44 ± 0.2 | - | 25 | This work |
| Clathrin-mCherry (vesicles) | OK | *i*MSD | - | 0.23 ± 0.06 | 25 | This work |
| H2B-EGFP | CHO-K1 | *i*MSD | 0.01 ± 0.02 | - | 25 | This work |

*The value was corrected for the wrong diffusion coefficient of the reference standard used in Schenk et al.

NA: not available

**Table S2.** Summary of the SPIM data acquisition parameters.

| Molecule | Sample | Image size (pixels) | Number of frames | Exposure time (ms) | Pixel size (nm) | Excitation power (μW) |
| --- | --- | --- | --- | --- | --- | --- |
| *In vitro* | | | | | | |
| Rhodamine110 | Water | 64 × 64 | 2,048 | 8 | 736* | 700 |
| EGFP | Water | 64 × 64 | 2,048 | 8 | 736* | 700 |
| 20-nm beads | Water | 64 × 64 | 2,048 | 8 | 736* | 150 |
| *In vivo* | | | | | | |
| EGFP | CHO-K1 | 64 × 64 | 8,192 | 4.5 | 184 | 100­­‒150 |
| EGFP-paxillin | CHO-K1 | 128 × 128 | 4,096 | 8 | 184 | 100­­‒150 |
| GR-RFP | CHO-K1 | 128 × 128 | 2,048 | 16 | 184 | 150‒250 |
| Lysosomes | CHO-K1 | 256 × 256 | 512 | 100 | 184 | 15­­‒30 |
| Clathrin-mCherry | OK | 256 × 256 | 2,048 | 16 | 184 | 70­­‒100 |
| H2B-EGFP | CHO-K1 | 256 × 256 | 2,048 | 16 | 184 | 100-150 |
| EGFP-paxillin-mCherry | CHO-K1 | 128 × 256 | 2,048 (each channel) | 8 | 184 | 100–250 |
| EGFP-paxillin & mCherry-paxillin | CHO-K1 | 128 × 256 | 2,048 (each channel) | 8 | 184 | 100–250 |

*Larger pixel sizes were obtained using 4×4 binning of the camera pixels.

**Supporting References**

1. Gendron, P.-O., Avaltroni, F. & Wilkinson, K.J. Diffusion Coefficients of Several Rhodamine Derivatives as Determined by Pulsed Field Gradient–Nuclear Magnetic Resonance and Fluorescence Correlation Spectroscopy. *J. Fluoresc.* **18**, 1093–1101 (2008).

2. Petrasek, Z. & Schwille, P. Precise measurement of diffusion coefficients using scanning fluorescence correlation spectroscopy. *Biophys. J.* **94**, 1437–1448 (2008).

3. Schenk, A., Ivanchenko, S., Rocker, C., Wiedenmann, J. & Nienhaus, G.U. Photodynamics of red fluorescent proteins studied by fluorescence correlation spectroscopy. *Biophys. J.* **86**, 384–394 (2004).

4. Digman, M.A. *et al.* Fluctuation correlation spectroscopy with a laser-scanning microscope: exploiting the hidden time structure. *Biophys. J.* **88**, L33–36 (2005).

5. Digman, M.A. *et al.* Measuring fast dynamics in solutions and cells with a laser scanning microscope. *Biophys. J.* **89**, 1317–1327 (2005).

6. Balint, S., Verdeny Vilanova, I., Sandoval Alvarez, A. & Lakadamyali, M. Correlative live-cell and superresolution microscopy reveals cargo transport dynamics at microtubule intersections. *Proc. Natl. Acad. Sci. USA* **110**, 3375–3380 (2013).

7. Sprague, B.L. *et al.* Analysis of binding at a single spatially localized cluster of binding sites by fluorescence recovery after photobleaching. *Biophys. J.* **91**, 1169–1191 (2006).
